# Patient Satisfaction and Long‐Term Outcomes: A 7‐ to 9‐Year Prospective Cohort Study of Root Canal Treatment in the Swedish Public Dental Service

**DOI:** 10.1111/iej.70018

**Published:** 2025-08-29

**Authors:** Emma Wigsten, Anita Afkhami, Hosaina Afewerki, Anna Levinsson, EndoReCo, Lars Bjørndal, Victoria S. Dawson, Helena Fransson, Fredrik Frisk, Peter Jonasson, Thomas Kvist, Merete Markvart, Maria Pigg, Dan Sebring, Emma Wigsten, Thomas Kvist

**Affiliations:** ^1^ Department of Endodontology, Institute of Odontology, The Sahlgrenska Academy University of Gothenburg Gothenburg Sweden; ^2^ Public Dental Service Region Västra Götaland Gothenburg Sweden; ^3^ Department of Epidemiology, Biostatistics and Occupational Health, School of Population and Global Health, Faculty of Medicine and Health Sciences McGill University Montreal Quebec Canada; ^4^ Division of Pediatric Dentistry, Department of Dental Medicine Karolinska Institutet Huddinge Sweden

**Keywords:** endodontics, general dental care, pain intensity, patient‐centred care, questionnaire, root canal therapy

## Abstract

**Aim:**

This prospective follow‐up study aimed to assess patient satisfaction with root canal treatment (RCT) 7–9 years after initiation in a general dental practice setting.

**Method:**

A study population of 243 patients initiated RCT at 20 public dental clinics in the Västra Götaland Region, Sweden. One to 3 years later, 159 patients (67.4%) responded to an 8‐item questionnaire assessing patient satisfaction with RCT and treatment results. The questionnaire was sent out again 7–9 years after treatment initiation. Descriptive and analytical statistics were used to compare respondents and non‐respondents, tooth groups and comparison over time.

**Results:**

A total of 156 patients (72.2%) responded to the 7–9 years' questionnaire; 82 women (52.6%) and 74 men (47.4%) with a mean age of 59.3 years (SD = ±15.3). Non‐respondents were significantly younger (*p* < 0.001). The majority of RCTs were reported as completed with a root filling (*n* = 102, 65.4%), although significantly fewer molars were completed (*n* = 43, 56.6%; *p* < 0.001). Sixty‐six patients (63.5%) reported no current pain, while most of those reporting pain described it as mild (*n* = 30, 90.9%). More than half of the root filled incisors were associated with current pain (*n* = 15, 57.7%; *p* < 0.009). A total of 111 patients (76.0%) recalled the procedure as painful. Chewing ability received the highest satisfaction rating (mean = 1.3). The majority of patients reported they would choose RCT again (*n* = 114, 77.0%). Among the 17 who answered ‘No’, 13 had undergone extraction, and 3 reported persistent pain following RCT. Over time, the number of extracted teeth increased (*p* < 0.001), while current pain intensity decreased and retrospective satisfaction with RCT improved (*p* < 0.001).

**Conclusions:**

Seven to nine years after the initiation of RCT in this general dental practice setting, patient satisfaction remains high despite one‐third of treated teeth being reported as extracted. These findings highlight the importance of incorporating patient‐reported outcomes in the evaluation of dental procedures, including endodontic treatments.

## 1 Introduction

Outcomes of root canal treatment (RCT) are commonly evaluated through a combination of clinical and radiographic evaluations. These typically focus on objective parameters such as tooth survival, absence of pain or discomfort and the status of the periapical tissues (Strindberg 1956; Ørstavik et al. 1986; Ng et al. 2007; Nixdorf et al. 2010; Ng et al. 2011a; Ng et al. 2011b). These parameters are often used by clinicians and researchers to determine whether the outcome of RCT constitutes a success or a failure (Strindberg 1956; Ørstavik et al. 1986; Ng et al. 2007; Nixdorf et al. 2010; Ng et al. 2011a; Ng et al. 2011b).

However, patients are often unaware of the persistent periapical inflammation due to its generally asymptomatic nature (Bergenholtz and Spångberg 2004; Friedman and Mor 2004). This may lead to discrepancies between the clinician's and the patient's subjective perception of treatment outcome (Reit and Kvist 1998; Atmeh and Hamasha 2020). Incorporating patient‐reported outcome measures alongside clinical and radiological evaluations may therefore provide a more comprehensive and patient‐centred assessment of RCT outcomes (Wigsten et al. 2021; Wigsten et al. 2022).

Patients' experiences are often explored within the concept of quality of life and satisfaction (Newsome and Wright 1999a, 1999b; Leong and Yap 2020). Today, these aspects are recognised as core outcomes across all endodontic treatment modalities, as highlighted by the European Society of Endodontology (ESE; El Karim et al. 2024).

Notably, most studies evaluating patient perspectives on RCT have been conducted in controlled settings such as universities, dental hospitals or specialist clinics (Gõrduysus and Gõrduysus 2000; Dugas et al. 2002; Gatten et al. 2011; Hamasha and Hatiwsh 2013; Torabinejad et al. 2014). However, as RCT is primarily performed in general dental practice (Fransson et al. 2016), there is a significant gap in the literature regarding patients' experiences in this setting (Wigsten et al. 2020; Wigsten et al. 2021). Furthermore, the predominance of cross‐sectional study designs highlights the need for prospective longitudinal studies (Hamasha and Hatiwsh 2013; Torabinejad et al. 2014).

This prospective follow‐up study aimed to assess patient satisfaction with RCT 7–9 years after the treatment initiation in a general dental practice setting.

## 2 Materials and Methods

The study adhered to the STROBE guidelines for cohort studies. The authors declare no conflict of interests. Written informed consent was obtained from all participants. Ethical approval for the study was provided by the Regional Ethical Committee in Gothenburg, Sweden, in 2015 (dnr: 857‐14). No monetary incentives were offered for participation in the study.

### 2.1 Study Design and Population

**FIGURE 1:**
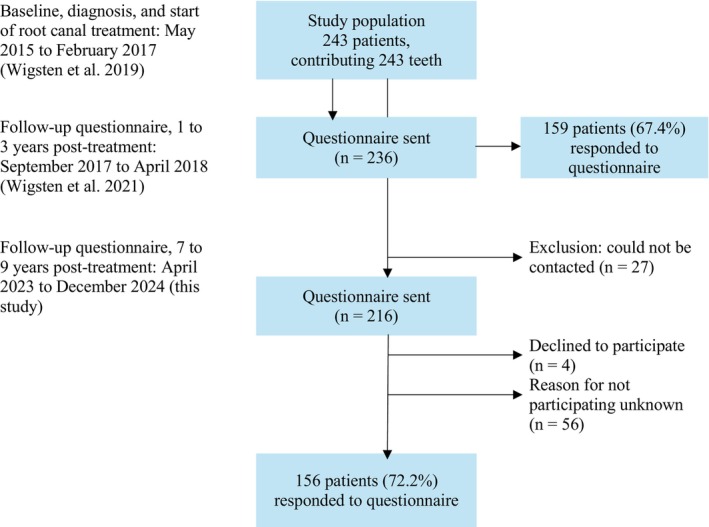
Flow chart of the study population. A total of 243 patients started root canal treatment during a designated 8‐week period at 20 public dental clinics in Sweden. Patient‐ and tooth‐specific characteristics were described in a previous study (Wigsten et al. 2019). One hundred fifty‐nine patients responded to the first questionnaire 1–3 years after treatment initiation, yielding a response rate of 67.4% (Wigsten et al. 2021). One hundred fifty‐six patients responded to the follow‐up questionnaire 7–9 years after treatment initiation, with a response rate of 72.2%.

The study included 243 patients who started RCT at 20 general dental care clinics within the public dental service in the Västra Götaland Region, Sweden, between 2015 and 2017 (Figure 1). Detailed patient‐ and tooth‐specific characteristics have been described in Wigsten et al. (2019). In short, the cohort included 128 women (52.7%) and 115 men (47.3%), aged 18–88 years (mean age 48.3 years, SD = ±16.4). The majority of RCTs were initiated on symptomatic teeth (*n* = 152, 64.9%), with the most common diagnosis being pulpal necrosis (*n* = 111, 47.0%). Molar teeth were the most frequently treated (*n* = 116, 47.7%).

One to 3 years after RCT initiation, 159 patients (67.4%) responded to a questionnaire on patient satisfaction and RCT outcomes (Wigsten et al. 2021; Figure 1). Reliability of the responses was considered good (weighted Kappa = 0.7184–0.9327; intraclass correlation coefficient = 0.540–0.796). In this follow‐up study, 7–9 years after starting RCT, two exclusion criteria were applied: missing contact details or a declination to participate in the study (Figure 1).

### 2.2 Questionnaire Design and Administration

A cover letter was attached to the questionnaire, providing a brief reminder about the study, instructions regarding the treated tooth and contact details for the research group. A pre‐addressed return envelope was included to facilitate the response. Non‐respondents received a reminder 3 weeks later, followed by a phone call. The distributions of questionnaires took place between April 2023 and December 2024 (Figure 1), with all materials presented in Swedish.

The questionnaire, detailed in Table 1, consisted of eight questions. Three of these questions were multiple‐choice (Questions 1, 3 and 8), while the remaining questions used visual analogue scales (VAS) with predefined endpoints. The VAS scales were 10 cm in length, ranging from ‘0’ (positive) to ‘10’ (negative).

**TABLE 1:** Overview of the questionnaire used to assess patients' perceptions of the outcomes of their root canal treatment.

Questions			
1	What has happened to the tooth?	□	The tooth is root filled
		□	The root canal treatment is not yet completed
		□	The tooth has been extracted and has been, or has not been, replaced

2	Mark how the tooth feels today:	No pain or discomfort	|‐‐‐‐‐‐‐‐‐‐‐|	Pain as bad as it could be

3	If you experience symptoms from the tooth, choose the option/s that best describe the character:					
	Burning sensation	Dull/Achy	Tender	Numbness	(electric) Shooting	Sharp
	Stabbing	Throbbing	Feels too high	Swelling	Pricking	Sensitive to touch

4	I experienced the root canal treatment as:	Not at all painful	|‐‐‐‐‐‐‐‐‐‐‐|	Very painful
5	I feel that my tooth looks:	Very good	|‐‐‐‐‐‐‐‐‐‐‐|	Very bad
6	I feel that chewing on the tooth is:	Unhindered	|‐‐‐‐‐‐‐‐‐‐‐|	Impossible
7	I feel that the root canal treatment was:	Absolutely worth the cost	|‐‐‐‐‐‐‐‐‐‐‐|	Absolutely not worth the cost

8	In retrospect would you have chosen root canal treatment for this tooth?	□	Yes
		□	No
		□	Uncertain

*Note:* The questionnaire has been translated into English, and its layout has been modified to be suitable for publication.

The first question addressed the status of the tooth. Questions 2 and 3 assessed current pain intensity and its characteristics. The subsequent four questions evaluated the patient's perspective, including their recollection of pain during RCT and their perceptions of aesthetics, function and costs. The final question (Question 8) asked whether the patient would, in hindsight, choose the same treatment again. Patients who reported that the tooth was missing were instructed to respond only to Questions 4 and 8.

### 2.3 Analysis of the Study Population

The questionnaires were labelled with each patient's unique identification number, allocated in the first study (Wigsten et al. 2019) to ensure the anonymity of the responses. All data were manually entered into an Excel datasheet (Microsoft Corp, Redmond, WA, USA). Pre‐operative patient‐ and tooth‐specific characteristics (Wigsten et al. 2019) and responses from the previous questionnaire study (Wigsten et al. 2021) were already registered. When data for a variable were absent, the corresponding cells in the datasheet were left empty and designated in the analysis as missing. The interval between baseline and the completion of the second questionnaire was registered.

### 2.4 Statistical Analysis

Descriptive statistics were presented as frequency and percentage, or as mean, standard deviation (SD), median, minimum and maximum as appropriate. The distribution of pre‐operative patient‐ and tooth‐specific characteristics was compared between respondents and non‐respondents to the 7–9 years questionnaire using chi‐squared test (categorical variables) and t‐test (continuous variables).

Analogously, chi‐squared test and ANOVA were used to compare the distribution of characteristics among respondents by tooth group as well as test for differences in patient satisfaction and treatment outcomes by tooth group. Finally, the change in patient satisfaction and treatment outcomes between the 1–3 years and the 7–9 years follow‐up among participants having responded to both questionnaires was tested using the chi‐squared test and paired t‐tests, with standardised mean difference presented as Cohen's d, using the sample SD of the mean difference as standardiser. All tests of statistical significance were two‐sided and conducted at a 5% significance level. The analyses were carried out using R (*version 4.2.1; R Foundation for Statistical Computing, Vienna, Austria*) and *IBM SPSS Statistics* (*version 28.0.1.1; IBM Corp., Armonk, NY, USA*).

## 3 Results

At the 7‐ to 9‐year follow‐up, 156 of 216 eligible patients responded to the questionnaire, representing a 72.2% response rate (Figure 1; Table 2). Twenty‐seven were excluded due to inability to contact. The mean time from RCT initiation to follow‐up was 8 years (92.9 months, SD = ±6.1; Table 3). A majority of respondents (*n* = 125, 80.1%) had also responded to the initial questionnaire distributed 1–3 years after treatment initiation (Wigsten et al. 2021).

**TABLE 2:** Patient‐ and tooth‐specific characteristics of the study population (*n* = 216), with a comparison between respondents (*n* = 156, 72.2%) and non‐respondents (*n* = 77, 27.8%) 7–9 years after initiation of root canal treatment in general dental practice.

Variable	Total	Respondents	Non‐respondents	*p*
	*n* = 216^a^	*n* = 156	*n* = 60	
Gender				0.853
Male	104 (48.1%)	74 (47.4%)	30 (50%)	
Female	112 (51.9%)	82 (52.6%)	30 (50%)	
Age at treatment				
Mean (SD)	48.9 (15.3)	51.6 (15.3)	41.9 (12.7)	< 0.001
Median (min; max)	50.0 (19.0; 84.0)	52.5 (19.0; 84.0)	41.0 (19.0; 72.0)	
Age group at treatment				< 0.001
Below 30 years old	28 (13.0%)	17 (10.9%)	11 (18.3%)	
30–39 years old	34 (15.7%)	18 (11.5%)	16 (26.7%)	
40–49 years old	45 (20.8%)	27 (17.3%)	18 (30.0%)	
50–59 years old	53 (24.5%)	45 (28.8%)	8 (13.3%)	
60–70 years old	37 (17.1%)	31 (19.9%)	6 (10%)	
Over 70 years old	19 (8.8%)	18 (11.5%)	1 (1.7%)	
Jaw				0.281
Maxilla	126 (58.3%)	95 (60.9%)	31 (51.7%)	
Mandible	90 (41.7%)	61 (39.1%)	29 (48.3%)	
Tooth type				0.957
Incisor	41 (19.0%)	29 (18.6%)	12 (20.0%)	
Premolar	71 (32.9%)	51 (32.7%)	20 (33.3%)	
Molar	104 (48.1%)	76 (48.7%)	28 (46.7%)	
Tooth type by jaw				0.248
Incisor (maxillary)	34 (15.7%)	23 (14.7%)	11 (18.3%)	
Incisor (mandibular)	7 (3.2%)	6 (3.8%)	1 (1.7%)	
Premolar (maxillary)	50 (23.1%)	36 (23.1%)	14 (23.3%)	
Premolar (mandibular)	21 (9.7%)	15 (9.6%)	6 (10%)	
Molar (maxillary)	42 (19.4%)	36 (23.1%)	6 (10%)	
Molar (mandibular)	62 (28.7%)	40 (25.6%)	22 (36.7%)	
Presence of pain^b^				0.883
Absence	70 (32.4%)	51 (32.7%)	19 (31.7%)	
Presence	137 (63.4%)	97 (62.2%)	40 (66.7%)	
Missing responses	9 (42.0%)	8 (5.1%)	1 (1.7%)	

*Note:* For categorical variables *n* (%) is presented.

^a^ 27 patients were not posted the questionnaire due to exclusion criteria being deceased or current address unknown.

^b^ The diagnosis on initiation of root canal treatment was registered by the general dental practitioners at start of the study (Wigsten et al. 2019). The indication was categorised based on the absence or presence of pain. Nine cases were missing.

**TABLE 3:** Characteristics of respondents (*n* = 156) distributed by tooth groups at baseline and at 7–9 years follow‐up in general dental practice.

Variable	Incisor	Premolar	Molar	*p*
	*n* = 29 (18.6%)	*n* = 51 (32.7%)	*n* = 76 (48.7%)	
Time between treatment and follow‐up (months)				
Mean (SD)	92.0 (5.6)	93.2 (6.5)	93.6 (6.3)	0.491
Median (min; max)	91.0 (79.0; 108.0)	94.0 (74.0; 109.0)	95.0 (83.0; 107.0)	
Gender				0.994
Male	14 (48.3%)	24 (47.1%)	36 (47.4%)	
Female	15 (51.7%)	27 (52.9%)	40 (52.6%)	
Age at treatment (years)				
Mean (SD)	49.9 (21.9)	55.5 (13.5)	49.6 (13.1)	0.054
Median (min; max)	58.0 (20.0; 81.0)	56.0 (25.0; 84.0)	51.0 (19.0; 79.0)	
Age group at treatment				< 0.002
Below 30 years old	7 (24.1%)	3 (5.9%)	7 (9.2%)	
30–39 years old	5 (17.2%)	4 (7.8%)	9 (11.8%)	
40–49 years old	1 (3.4%)	8 (15.7%)	18 (23.7%)	
50–59 years old	2 (6.9%)	17 (33.3%)	26 (34.2%)	
60–70 years old	7 (24.1%)	11 (21.6%)	13 (17.1%)	
Over 70 years old	7 (24.1%)	8 (15.7%)	3 (3.9%)	
Age at follow‐up (years)				
Mean (SD)	57.6 (21.9)	63.3 (13.4)	57.3 (13.1)	0.048
Median (min; max)	66.0 (28.0; 89.0)	63.0 (33.0; 92.0)	59.0 (27.0; 86.0)	
Below 30 years old	4 (13.8%)	0 (0%)	2 (2.6%)	< 0.001
30–39 years old	6 (20.7%)	3 (5.9%)	7 (9.2%)	
40–49 years old	2 (6.9%)	5 (9.8%)	12 (15.8%)	
50–59 years old	1 (3.4%)	12 (23.5%)	20 (26.3%)	
60–70 years old	3 (10.3%)	14 (27.5%)	23 (30.3%)	
Over 70 years old	13 (44.8%)	17 (33.3%)	12 (15.8%)	
Jaw				0.003
Maxilla	23 (79.3%)	36 (70.6%)	36 (47.4%)	
Mandible	6 (20.7%)	15 (29.4%)	40 (52.6%)	
Presence of pain^a^				0.231
Absence	13 (44.8%)	13 (25.5%)	25 (32.9%)	
Presence	15 (51.7%)	35 (68.6%)	47 (61.8%)	
Missing response	1 (3.4%)	3 (5.9%)	4 (5.3%)	

*Note:* For categorical variables *n* (%) is presented.

^a^ The diagnosis on initiation of root canal treatment was registered by the general dental practitioners at the start of the study (Wigsten et al. 2019). The indication was categorised based on the absence or presence of pain. Nine cases were missing, of which eight were among the 7‐ to 9‐year follow‐up questionnaire respondents.

### 3.1 Respondents and Non‐Respondents

Eighty‐two women (52.6%) and 74 men (47.4%) responded (Table 2). The mean age was 59.3 years (SD = ±15.3). Attrition analysis showed that non‐respondents were significantly younger, with a mean age of 41.9 years (SD = ±12.7) versus 51.6 years (SD = ±15.3; *p* < 0.001).

### 3.2 Patient and Tooth Characteristics by Tooth Groups

Molars were the most commonly treated tooth group (*n* = 76, 48.7%), followed by premolars (*n* = 51, 32.7%) and incisors/canines (*n* = 29, 18.6%; Table 3). Most incisors and premolars were in the maxilla, while molars were more evenly distributed between the jaws (*p* = 0.003). Nearly a quarter of the incisor group patients were aged under 30, compared to 6% and 9% in the premolar and molar groups, respectively (*p* = 0.002). RCT had commonly been initiated due to pain (*n* = 97, 62.2%).

### 3.3 Treatment Completion After Initiated Root Canal Treatment

The majority of respondents reported a completed RCT (*n* = 102, 65.4%; Table 4), and 52 (33.4%) reported that the tooth had been extracted. A significantly larger proportion of incisors (89.7%) were reported as completed, compared to 64.7% of premolars and 56.6% of molars (*p* < 0.001).

**TABLE 4:** Evaluating of patient satisfaction with root canal treatment and its outcomes, including comparisons between tooth groups.

Variable	Total (*n* = 156)	Incisor (*n* = 29)	Premolar (*n* = 51)	Molar (*n* = 76)	*p*
Treatment completion					< 0.001
Root filled	102 (65.4%)	26 (89.7%)	33 (64.7%)	43 (56.6%)	
Non‐root filled	0 (0%)	0 (0%)	0 (0%)	0 (0%)	
Extracted	52 (33.4%)	3 (10.3%)	17 (33.3%)	32 (42.1%)	
Missing responses	2 (1.3%)	0 (0%)	1 (2%)	1 (1.3%)	
Present pain intensity					
Mean (SD)	0.6 (1.3)	0.8 (0.9)	0.9 (2.0)	0.4 (0.6)	0.207
Median (min; max)	0.2 (0.0; 8.7)	0.5 (0.0; 3.8)	0.2 (0.0; 8.7)	0.2 (0.0; 3.2)	
No pain^a^	66 (63.5%)	11 (42.3%)	22 (64.7%)	33 (75.0%)	0.009
Mild pain	30 (28.8%)	14 (53.8%)	7 (20.6%)	9 (20.5%)	
Moderate pain	1 (1.0%)	1 (3.8%)	0 (0%)	0 (0%)	
Severe pain	2 (1.9%)	0 (0%)	2 (5.9%)	0 (0%)	
Missing responses^b^	5 (4.8%)	0 (0%)	3 (8.8%)	2 (4.5%)	
Intra‐operative pain					
Mean (SD)	2.7 (2.5)	2.0 (2.4)	2.7 (2.3)	2.9 (2.6)	0.221
Median (min; max)	2.0 (0.0; 9.5)	0.75 (0.0; 7.5)	2.3 (0.0; 9.5)	2.45 (0.0; 9.4)	
No pain^a^	35 (22.4%)	11 (37.9%)	9 (17.6%)	15 (19.7%)	0.363
Mild pain	61 (39.1%)	10 (34.5%)	23 (45.1%)	28 (36.8%)	
Moderate pain	36 (23.1%)	4 (13.8%)	13 (25.5%)	19 (25%)	
Severe pain	14 (9.0%)	3 (10.3%)	3 (5.9%)	8 (10.5%)	
Missing responses	10 (6.4%)	1 (3.4%)	3 (5.9%)	6 (7.9%)	
Post‐operative aesthetics					
Mean (SD)	2.0 (2.2)	2.7 (2.7)	1.8 (2.2)	1.7 (1.7)	0.202
Median (min; max)	0.90 (0.0; 10.0)	1.35 (0.0; 8.1)	0.8 (0.0; 10.0)	0.9 (0.0; 5.4)	
Missing responses^b^	13 (12.5%)	2 (6.9%)	3 (5.9%)	8 (10.5%)	
Chewing ability					
Mean (SD)	1.3 (1.8)	1.4 (2.1)	1.5 (2.0)	1.1 (1.4)	0.615
Median (min; max)	0.5 (0.0; 8.2)	0.45 (0.0; 8.2)	0.55 (0.0; 7.6)	0.45 (0.0; 5.2)	
Missing responses^b^	10 (9.6%)	2 (6.9%)	2 (3.9%)	6 (7.9%)	
Cost					
Mean (SD)	1.6 (2.1)	1.6 (1.8)	1.8 (2.6)	1.3 (1.7)	0.643
Median (min; max)	0.5 (0.0; 10.0)	0.7 (0.0; 5.2)	0.4 (0.0; 10.0)	0.5 (0.0; 5.7)	
Missing responses^b^	16 (15.4%)	5 (17.2%)	4 (7.8%)	7 (9.2%)	
RCT in retrospect					
Yes	114 (73.1%)	26 (89.7%)	38 (74.5%)	50 (65.8%)	0.132
No	17 (10.9%)	1 (3.4%)	7 (13.7%)	9 (11.8%)	
Uncertain	17 (10.9%)	1 (3.4%)	4 (7.8%)	12 (15.8%)	
Missing responses	8 (5.1%)	1 (3.4%)	2 (3.9%)	5 (6.6%)	

*Note:* For categorical variables *n* (%) is presented.

^a^ Present and intra‐operative pain measurements were categorised as follows: no pain = 0.0–0.4; mild pain = 0.5–3.4, moderate pain = 3.5–6.4; severe pain = 6.5–10.0.

^b^ Patients who reported that the tooth had been extracted were only asked to estimate ‘intra‐operative pain’ and ‘RCT in retrospect’.

### 3.4 Present Pain Intensity and Descriptive Pain Characteristics After Root Canal Treatment

Out of the 102 patients with completed RCT, 66 patients (63.5%) reported no current pain or discomfort (VAS = 0; Table 4). Among patients reporting current pain, the majority indicated mild pain (*n* = 30, 90.9%, VAS 0.1–3.0). Three patients reported moderate or severe pain (2.9%). The mean pain intensity was 0.6 (SD = ±1.3). Reported current pain differed significantly across tooth types: 57.7% of incisors (*n* = 15), 29.0% of premolars (*n* = 9) and 21.4% of molars (*n* = 9; *p* < 0.009).

Thirteen patients reported one or more pain characteristics (total 17 characteristics), with the affected teeth being 6 incisors, 1 premolar and 6 molars. The three most common characteristics were tenderness to touch (*n* = 6, 35.3%), sharp pain (*n* = 3, 17.6%) and dull/achy pain (*n* = 3, 17.6%). The majority reported mild pain (*n* = 9, 69.2%), while two (15.4%) reported moderate and one (7.7%) severe pain. The mean pain intensity was 2.2 (SD = ±2.1, median = 1.7).

### 3.5 Recollection of Intraoperative Pain During Root Canal Treatment

Thirty‐five patients (24.0%) reported no memory of pain or discomfort during the procedure (VAS = 0; Table 4). The remaining 111 respondents (76.0%) reported pain or discomfort, with the majority indicating mild pain (*n* = 61, 55.0%, VAS 0.1–3). The mean pain intensity was 2.7 (SD = ±2.5), with no significant differences between the tooth groups.

### 3.6 Patient Satisfaction With Aesthetics, Chewing Ability and Treatment Costs

Mean values of respondents' perceptions of aesthetics, chewing ability and costs ranged from VAS 1.6 to 2.0, with lower ratings indicating greater satisfaction (Table 4). The majority scored within the ‘0–3’ range for all three items: 68 (74.7%) for aesthetics, 79 (84.0%) for chewing ability and 70 (79.5%) for costs. No significant differences were observed between the tooth groups.

### 3.7 In Retrospect: Patient Perspectives on Undergoing Root Canal Treatment After 7–9 Years

The majority of patients (*n* = 114, 77.0%) reported they would choose to undergo RCT again (Table 4). Seventeen patients (11.5%) responded with either ‘No’ or were ‘Uncertain’. No significant differences were observed between the tooth groups (*p* = 0.132).

There were no statistically significant differences in baseline patient‐ or tooth‐specific characteristics between those retrospectively willing to undergo RCT or not (Table 6). Among patients positive towards repeating RCT, the majority (*n* = 89, 78.1%) had their tooth preserved and root filled, whereas among those unwilling to repeat RCT, the majority had their tooth extracted (*n* = 23, 67.7%; *p* < 0.001). Overall, patients willing to repeat the procedure reported less intra‐operative pain compared to those unwilling to repeat RCT (*p* = 0.019). Furthermore, present pain experience differed significantly between patients who would repeat RCT and those who would not (*p* = 0.006); of the 93 patients reporting no or mild pain, 85 (91.4%) registered that they would undergo RCT again. Greater satisfaction with aesthetic outcomes, chewing ability and treatment cost was also associated with a higher likelihood of retrospective willingness to undergo RCT (Table 6).

### 3.8 Comparing Respondents' Ratings at the 1‐ to 3‐Year and 7‐ to 9‐Year Follow‐Ups

Among patients who responded to both questionnaires, the proportion of extracted teeth increased significantly between follow‐ups, while the number of root filled teeth decreased at follow‐up (*p* < 0.001; Table 5).

**TABLE 5:** Comparison of responses from 125 patients who responded the questionnaire at both the 1‐ to 3‐year and 7‐ to 9‐year follow‐ups.

Variable	1‐ to 3‐year follow‐up	7‐ to 9‐year follow‐up	Standardised mean difference	*p*
Treatment completion^a^				
Root filled	87 (69.6%)	81 (64.8%)		< 0.001
Non‐root filled	7 (5.6%)	0 (0%)		
Extracted	31 (24.8%)	43 (34.4%)		
Missing responses	0 (0%)	1 (0.8%)		

	*n*	Mean (SD)	Mean (SD)	Cohen's *d* (95% CI)^b^	*p*
Present pain intensity	68	1.5 (2.2)	0.6 (1.0)	0.5 (0.20–0.70)	< 0.001
Intraoperative pain	115	2.5 (2.4)	2.6 (2.3)	−0.1 (−0.24–0.13)	0.540
Post‐operative aesthetics	61	1.9 (1.8)	1.9 (2.2)	0.0 (−0.25–0.26)	0.960
Chewing ability	63	1.6 (1.8)	1.2 (1.6)	0.2 (−0.05–0.45)	0.121
Cost	59	1.9 (2.3)	1.4 (2.0)	0.3 (0.00–0.52)	0.051
RCT in retrospect^a^					
Yes		90 (72.0%)	93 (74.4%)		< 0.001
No		12 (9.6%)	14 (11.2%)		
Uncertain		15 (12.0%)	10 (8.0%)		
Missing responses		8 (6.4%)	8 (6.4%)		

^a^ For categorical variables *n* (%) is presented.

^b^ Cohen's *d* uses the sample standard deviation of the mean difference.

**TABLE 6:** Patient‐ and tooth‐specific characteristics and satisfaction outcomes in comparison to willingness to undergo root canal treatment in retrospect at 7–9 years follow‐up. Comparison between the responses ‘Yes’, ‘No’ and ‘Uncertain’.

Variable	Yes	No	Uncertain	*p*	Yes	No/uncertain	*p*
	(*n* = 114)	(*n* = 17)	(*n* = 17)		(*n* = 114)	(*n* = 34)	
Patient‐ and tooth‐specific characteristics							
Gender				0.788			1.000
Male	53 (46.5%)	9 (52.9%)	7 (41.2%)		53 (46.5%)	16 (47.1%)	
Female	61 (53.5%)	8 (47.1%)	10 (58.8%)		61 (53.5%)	18 (52.9%)	
Age at treatment				0.655			0.626
Mean (SD)	51.8 (15.9)	52.4 (13.1)	48.3 (13.0)		51.8 (15.9)	50.4 (13.0)	
Age group at treatment				0.407			0.117
Below 30 years old	14 (12.3%)	1 (5.9%)	1 (5.9%)		14 (12.3%)	2 (5.9%)	
30–39 years old	15 (13.2%)	1 (5.9%)	2 (11.8%)		15 (13.2%)	3 (8.8%)	
40–49 years old	15 (13.2%)	6 (35.3%)	4 (23.5%)		15 (13.2%)	10 (29.4%)	
50–59 years old	32 (28.1%)	5 (29.4%)	7 (41.2%)		32 (28.1%)	12 (35.3%)	
60–69 years old	22 (19.3%)	3 (17.6%)	3 (17.6%)		22 (19.3%)	6 (17.6%)	
Over 70 years old	16 (14.0%)	1 (5.9%)	0 (0%)		16 (14.0%)	1 (2.9%)	
Jaw				0.964			0.944
Maxilla	70 (61.4%)	10 (58.8%)	10 (58.8%)		70 (61.4%)	20 (58.8%)	
Mandible	44 (38.6%)	7 (41.2%)	7 (41.2%)		44 (38.6%)	14 (41.2%)	
Tooth group				0.132			0.057
Incisor	26 (22.8%)	1 (5.9%)	1 (5.9%)		26 (22.8%)	2 (5.9%)	
Premolar	38 (33.3%)	7 (41.2%)	4 (23.5%)		38 (33.3%)	11 (32.4%)	
Molar	50 (43.9%)	9 (52.9%)	12 (70.6%)		50 (43.9%)	21 (61.8%)	
Tooth type by jaw				0.596			0.265
Incisor (maxillary)	20 (17.5%)	1 (5.9%)	1 (5.9%)		20 (17.5%)	2 (5.9%)	
Incisor (mandibular)	6 (5.3%)	0 (0%)	0 (0%)		6 (5.3%)	0 (0%)	
Premolar (maxillary)	27 (23.7%)	4 (23.5%)	3 (17.6%)		27 (23.7%)	7 (20.6%)	
Premolar (mandibular)	11 (9.6%)	3 (17.6%)	1 (5.9%)		11 (9.6%)	4 (11.8%)	
Molar (maxillary)	23 (20.2%)	5 (29.4%)	6 (35.3%)		23 (20.2%)	11 (32.4%)	
Molar (mandibular)	27 (23.7%)	4 (23.5%)	6 (35.3%)		27 (23.7%)	10 (29.4%)	
Presence of pain^a^				0.273			0.411
Absence	39 (34.2%)	6 (35.3%)	2 (11.8%)		39 (34.2%)	8 (23.5%)	
Presence	70 (61.4%)	11 (64.7%)	12 (70.6%)		70 (61.4%)	23 (67.6%)	
Missing responses	5 (4.4%)	0 (0%)	3 (17.6%)		5 (4.4%)	3 (8.8%)	

Variable	Yes	No	Uncertain	*p*	Yes	No/uncertain	*p*
	(*n* = 114)	(*n* = 17)	(*n* = 17)		(*n* = 114)	(*n* = 34)	
Satisfaction with root canal treatment and its outcomes							
Treatment completion				< 0.001			< 0.001
Root filled	89 (78.1%)	3 (17.6%)	7 (41.2%)		89 (78.1%)	10 (29.4%)	
Non‐root filled	0 (0%)	0 (0%)	0 (0%)		0 (0%)	0 (0%)	
Extracted	15 (13.2%)	10 (58.8%)	6 (35.3%)		15 (13.2%)	16 (47.1%)	
Extracted and replaced	10 (8.8%)	3 (17.6%)	4 (23.5%)		10 (8.8%)	7 (20.6%)	
Missing responses	0 (0%)	1 (5.9%)	0 (0%)		0 (0%)	1 (2.9%)	
Present pain intensity				< 0.001			0.006
No pain	59 (51.8%)	0 (0%)	5 (29.4%)		59 (51.8%)	5 (14.7%)	
Mild pain	26 (22.8%)	2 (11.8%)	1 (5.9%)		26 (22.8%)	3 (8.8%)	
Moderate pain	0 (0%)	0 (0%)	1 (5.9%)		0 (0%)	1 (2.9%)	
Severe pain	1 (0.9%)	1 (5.9%)	0 (0%)		1 (0.9%)	1 (2.9%)	
Missing responses	28 (24.6%)	14 (82.4%)	10 (58.8%)		28 (24.6%)	24 (70.6%)	
Intraoperative pain				0.09			0.019
No pain	30 (26.3%)	2 (11.8%)	1 (5.9%)		30 (26.3%)	3 (8.8%)	
Mild pain	50 (43.9%)	5 (29.4%)	5 (29.4%)		50 (43.9%)	10 (29.4%)	
Moderate pain	22 (19.3%)	5 (29.4%)	7 (41.2%)		22 (19.3%)	12 (35.3%)	
Severe pain	8 (7%)	3 (17.6%)	2 (11.8%)		8 (7.0%)	5 (14.7%)	
Missing responses	4 (3.5%)	2 (11.8%)	2 (11.8%)		4 (3.5%)	4 (11.8%)	
Post‐operative aesthetics				< 0.001			0.11
Mean (SD)	1.8 (1.9)	8.1 (1.8)	1.8 (1.6)		1.8 (1.9)	3.9 (3.5)	
Chewing ability				< 0.001			< 0.001
Mean (SD)	1.0 (1.6)	5.9 (0.9)	2.5 (1.6)		1.0 (1.6)	3.6 (2.2)	
Cost				0.102			0.029
Mean (SD)	1.2 (1.6)	7.4 (4.0)	2.8 (2.1)		1.2 (1.6)	4.3 (3.5)	

*Note:* For categorical variables *n* (%) is presented.

^a^ The diagnosis on initiation of root canal treatment was registered by the general dental practitioners at the start of the study (Wigsten et al. 2019). The indication was categorised based on the absence or presence of pain. Eight cases were missing.

Pain intensity decreased significantly between follow‐ups with a medium effect size (d = 0.5, 95% CI 0.20–0.70). Estimated treatment cost satisfaction showed a slight improvement (small effect size d = 0.3, 95% CI 0.00–0.52; Table 5).

Patients' attitudes towards RCT changed significantly, with more reporting that they would have chosen RCT in retrospect (*p* < 0.001; Table 5).

## 4 Discussion

Among the 156 patients available for follow‐up after 7–9 years (72.2%) in this prospective study, 102 teeth (65.4%) were root filled, while 52 teeth (33.4%) were extracted.

### 4.1 Tooth Extraction Following Initiation of Root Canal Treatment

Follow‐up studies commonly define baseline at the completion of RCT with the permanent root filling (Strindberg 1956; Ng et al. 2007; Nixdorf et al. 2010; Ng et al. 2011a; Ng et al. 2011b). In contrast, our study defined baseline as the appointment at which a tooth was diagnosed with pulp and periradicular disease and RCT was initiated (Wigsten et al. 2019; Wigsten et al. 2021; Wigsten et al. 2022). Although this may result in a less favourable view on RCT as a therapy modality, it provides a more comprehensive perspective. The relatively high number of extracted teeth in our cohort is to a certain extent explained by the fact that among all originally included, 32 patients (13.3%) had their tooth extracted before completion of RCT (Wigsten et al. 2022).

In particular, the high frequency of extracted molars (42.1%) was of concern in this study (Table 4). These findings corroborate with the previous studies presenting 1‐ and 3‐year follow‐ups of the same cohort (Wigsten et al. 2021; Wigsten et al. 2022). The findings also align with other follow‐up studies on root filled teeth, in which molars consistently show lower survival rates compared to other tooth groups (Fransson et al. 2016; Fransson et al. 2021; Göransson et al. 2021; Kebke et al. 2021). The reasons behind these observations are not fully understood, but a combination of occlusal and articulatory forces, combined with an inadequate coronal restoration, is generally considered to be a part of the explanation (Ng et al. 2011b). This view is supported by data clearly demonstrating better survival rates for teeth restored with an indirect restoration (Fransson et al. 2021). Furthermore, molars repeatedly exhibit complexity and technical challenges during RCT (Peters 2016). In cross‐sectional studies, molars present a high prevalence of technically inadequate root fillings and consequently a higher prevalence of apical periodontitis (Eckerbom et al. 2007; Laukkanen et al. 2021; Silnovic et al. 2023). The persistence of apical periodontitis in root filled teeth has also been demonstrated to increase the risk of tooth extraction (Kirkevang et al. 2017; Olsson et al. 2024).

In contrast to the poor survival rate among molars, the number of extracted incisors (10.3%) was low. In addition to their less vulnerable tooth position, easier access for RCT and a lower prevalence of persistent apical periodontitis (Silnovic et al. 2023), their perceived aesthetic and functional value may be involved. As patients often consider them particularly important and prioritise their preservation, a suboptimal outcome in terms of periapical status and comfort may be accepted (Gatten et al. 2011; Olsson et al. 2024).

Although the reasons for extraction over the 7‐ to 9‐year period remain unknown, first‐year data with 240 teeth indicated that most extractions (*n* = 17, 54.8%) were associated with endodontic complications such as apical periodontitis, perforations, instrument fractures and dentinal cracks (Wigsten et al. 2022). Seven teeth (22.6%) were extracted due to extensive tooth substance loss. In the remaining cases (*n* = 7, 22.6%), the extractions were performed at the patient's request for various reasons. In one case, the reason for extraction were not documented. This aligns with findings from other studies in general practice, where fractures, endodontic failure and caries have been identified as the primary causes of extraction in root filled teeth (Göransson et al. 2021; Kebke et al. 2021).

### 4.2 Pain Intensity: Present and Recalled Pain

RCT is often initiated due to pain (Reit et al. 1993; Bjørndal et al. 2006; Wigsten et al. 2019). Over time, the majority of patients reported absence of pain (63.5%) as well as a reduction in pain intensity. Nevertheless, the frequency of pain reported in this study was higher than observed in previous studies. For example, Nixdorf et al. (2010) and Jonsson Sjögren et al. (2019) found that approximately 5% of patients experienced persistent pain or discomfort from their root filled teeth. In contrast, Polycarpou et al. (2005) reported a significantly higher prevalence of 21.1% in a cohort assessed through clinical and radiographic examination.

This discrepancy may partly be attributed to differences in study design, clinical settings and recruitment procedures. Prospective studies involving patients who have undergone RCT in general dental practice, and particularly with baseline at treatment start, are rare. Comparing the results of persistent pain from this study with those from cross‐sectional or retrospective studies is problematic.

In terms of tooth type, incisors were more frequently associated with post‐treatment pain compared to other tooth groups (*p* = 0.009). Although the underlying reason remains unclear, patient‐related factors such as age and gender may contribute. However, this finding is in line with the above presumption that mild pain may be tolerable in highly valued anterior teeth, which may protect them from extraction. Torabinejad et al. (1988) and Jonsson Sjögren et al. (2019) reported a higher prevalence of postoperative pain among younger individuals, while Polycarpou et al. (2005) identified several risk factors for chronic pain, including female gender, a history of painful treatments, present pain and pain duration, among others. Further research is needed to clarify the correlation between demographic parameters and long‐term pain following RCT.

Regarding the treatment procedure itself, the majority of patients (76.0%) perceived it as painful, which aligns with findings from previous studies (Leclaire et al. 1988; Segura‐Egea et al. 2009; Murillo‐Benítez et al. 2020). Segura‐Egea et al. (2009) reported a lower prevalence of pain (46%), with most cases classified as mild in intensity.

### 4.3 Patient Satisfaction and Willingness to Choose Root Canal Treatment Again

Despite the high frequency of reported pain, most patients expressed satisfaction with their RCT. While most research has been conducted in controlled settings such as universities or specialist clinics (Gõrduysus and Gõrduysus 2000; Dugas et al. 2002; Gatten et al. 2011; Hamasha and Hatiwsh 2013), the present study supports previous findings that RCT performed in general dental practice can also result in high levels of satisfaction.

Although cost is often reported as a source of dissatisfaction in other studies (Dugas et al. 2002; Gatten et al. 2011; Hamasha and Hatiwsh 2013), it was not identified as a significant concern among patients in this cohort. Furthermore, satisfaction levels were consistent across tooth groups.

Most patients (77.0%) reported they would choose the same procedure again. Similar findings have been reported in previous studies, where patients generally preferred to retain their natural dentition whenever possible. Many also indicated that they would recommend the treatment or choose it again if needed (Lobb et al. 1996; Gõrduysus and Gõrduysus 2000; Gatten et al. 2011).

However, patients who registered moderate to severe pain, either during treatment or at follow‐up, were significantly less likely to choose RCT again. This was particularly evident for teeth registered as extracted compared to those that had been preserved and root filled. Additionally, lower satisfaction with aesthetic outcomes, chewing ability and treatment costs was associated with reduced retrospective acceptance of RCT.

### 4.4 Strengths and Limitations of the Study

The primary strength of this study lies in its real‐world prospective design, involving patients recruited from general dental clinics. This enhances the relevance and generalisability of the findings to everyday clinical practice. Additionally, the baseline set at the start of treatment and the long follow‐up period (7–9 years) provide insight into both tooth survival and patient‐reported outcomes, such as persistent pain, recollection of pain during treatment and treatment acceptance (Statens Beredning för medicinsk Utvärdering 2010; Duncan et al. 2023).

However, the study was set up within a particular context, involving 20 Public Dental Service clinics in one region in Sweden. The respondents were significantly older, potentially due to the postal distribution method (Table 2). A mixed‐mode approach might have improved participation rates among younger patients (Edwards et al. 2002). Nevertheless, the response rate of 72.2% is considered good for long‐term follow‐up studies in general dental practice (Edwards et al. 2002).

Recall bias is a potential limitation due to the long interval between baseline and follow‐up. However, extended follow‐up provides insights into long‐term satisfaction, which may be unobserved in short‐term evaluations. Although recall bias cannot be entirely excluded, it is less likely to affect persistent symptoms or major treatment events (Stull et al. 2009). Global assessments, such as overall satisfaction, are also less vulnerable to recall bias than symptom‐specific reports (Stull et al. 2009). To improve recall accuracy, the questionnaire included a tooth illustration to aid correct identification. The reliability of the questionnaire was evaluated in a previous study (Wigsten et al. 2021).

Furthermore, neither the patients nor the dentists in this study may be representative of RCTs in other settings, either within Sweden or internationally. Patient satisfaction is formed by expectations influenced by sociocultural and systemic factors, including healthcare structure, access to care and cultural norms (Sitzia and Wood 1997; Newsome and Wright 1999a; Newsome and Wright 1999b). These factors should be considered when interpreting the generalisability of the results to other contexts.

The ESE clinical guidelines emphasise the importance of preserving natural teeth in case of need of RCT, as this prevents or delays the demands of prosthetic replacements (Duncan et al. 2023). Nonetheless, decision‐making about a tooth requiring RCT should be made on an individual basis, weighing both clinical and patient‐related factors.

While most initiated RCTs in this study were completed and patient satisfaction was high, outcome assessment remains incomplete without clinical and radiographic evaluation of the periapical status. The relatively high frequency of extractions is concerning and highlights the need to compare molar RCT outcomes with alternative treatment options (Savolainen et al. 2025).

We recommend that future randomised controlled trials on RCT specifically investigate whether treatment outcomes differ significantly depending on whether the procedure is performed by general practitioners or by dental specialists, within a single cohort and under a standardised study design.

However, studies directly comparing RCT with alternative interventions for severely compromised teeth involving the pulp and periapical tissues are also needed. Ideally, such comparisons should also be conducted as randomised controlled trials, including both clinical and patient‐reported outcomes. Although randomisation may be challenging due to the complexity of the situation and individualised nature of treatment decisions.

## 5 Conclusions

Seven to nine years after the initiation of RCT in this general practice setting, patient satisfaction remains high, despite one‐third of the treated teeth being reported as extracted. These findings highlight the importance of incorporating patient‐reported outcomes in the evaluation of various dental procedures, including endodontic treatments.

## Author Contributions

**Emma Wigsten:** conceptualization; data curation (lead); formal analysis; funding acquisition (lead); investigation (lead); methodology; project administration (lead); visualization; writing – original draft preparation (lead). **Anita Afkhami** and **Hosaina Afewerki:** investigation; visualization; writing – original draft preparation. **Anna Levinsson:** formal analysis (lead); validation; visualization; writing – review and editing. **Thomas Kvist:** conceptualization (lead); funding acquisition; methodology; supervision; writing – review and editing. All authors gave final approval and agreed to be accountable for all aspects of the work. The collaborators in EndoReCo have critically revised the manuscript.

## Ethics Statement

This study was approved by the Regional Ethical Committee in Gothenburg, Sweden (Dnr: 817‐16). The research has been conducted in full accordance with ethical principles, including the World Medical Association Declaration of Helsinki (version 2008) and the requirements of Swedish law, under which the research has been conducted. All participating patients have received written and oral information about the study and have provided their verbal and written informed consent. The data do not contain any information that could identify the participants.

## Conflicts of Interest

The authors declare no conflicts of interest.

## Data Availability

The data that support the findings of this study are available from the corresponding author, EW, upon reasonable request.

## References

[iej70018-bib-0001] Atmeh, A. , and A. A. Hamasha . 2020. “Outcome Assessment of Non‐Surgical Root Canal Treatment by Patients: What Factors Can Influence Their Evaluation?” British Dental Journal 228, no. 10: 762–766. 32444749 10.1038/s41415-020-1528-4

[iej70018-bib-0002] Bergenholtz, G. , and L. Spångberg . 2004. “Controversies in Endodontics.” Critical Reviews in Oral Biology & Medicine 15, no. 2: 99–114. 15059945 10.1177/154411130401500204

[iej70018-bib-0003] Bjørndal, L. , M. H. Laustsen , and C. Reit . 2006. “Root Canal Treatment in Denmark Is Most Often Carried out in Carious Vital Molar Teeth and Retreatments Are Rare.” International Endodontic Journal 39, no. 10: 785–790. 16948664 10.1111/j.1365-2591.2006.01149.x

[iej70018-bib-0004] Dugas, N. N. , H. P. Lawrence , P. Teplitsky , and S. Friedman . 2002. “Quality of Life and Satisfaction Outcomes of Endodontic Treatment.” Journal of Endodontics 28, no. 12: 819–827. 12489651 10.1097/00004770-200212000-00007

[iej70018-bib-0005] Duncan, H. F. , L.‐L. Kirkevang , O. A. Peters , et al. 2023. “Treatment of Pulpal and Apical Disease: The European Society of Endodontology (ESE) S3‐Level Clinical Practice Guideline.” International Endodontic Journal 56, no. Suppl 3: 238–295. 37772327 10.1111/iej.13974

[iej70018-bib-0006] Eckerbom, M. , L. Flygare , and T. Magnusson . 2007. “A 20‐Year Follow‐Up Study of Endodontic Variables and Apical Status in a Swedish Population.” International Endodontic Journal 40, no. 12: 940–948. 17883402 10.1111/j.1365-2591.2007.01290.x

[iej70018-bib-0007] Edwards, P. , I. Roberts , M. Clarke , et al. 2002. “Increasing Response Rates to Postal Questionnaires: Systematic Review.” British Medical Journal 324, no. 7347: 1183. 12016181 10.1136/bmj.324.7347.1183 PMC111107

[iej70018-bib-0008] El Karim, I. , H. F. Duncan , S. Cushley , et al. 2024. “An International Consensus Study to Identify “What” Outcomes Should Be Included in a Core Outcome Set for Endodontic Treatments (COSET) for Utilization in Clinical Practice and Research.” International Endodontic Journal 57, no. 3: 270–280. 38314586 10.1111/iej.14008

[iej70018-bib-0009] Fransson, H. , L. Bjørndal , F. Frisk , et al. 2021. “Factors Associated With Extraction Following Root Canal Filling in Adults.” Journal of Dental Research 100, no. 6: 608–614. 33402028 10.1177/0022034520982962 PMC8138334

[iej70018-bib-0010] Fransson, H. , V. S. Dawson , F. Frisk , L. Bjørndal , EndoReCo , and T. Kvist . 2016. “Survival of Root‐Filled Teeth in the Swedish Adult Population.” Journal of Endodontics 42, no. 2: 216–220. 26813417 10.1016/j.joen.2015.11.008

[iej70018-bib-0011] Friedman, S. , and C. Mor . 2004. “The Success of Endodontic Therapy‐‐Healing and Functionality.” Journal of the California Dental Association 32, no. 6: 493–503. 15344440

[iej70018-bib-0012] Gatten, D. L. , C. A. Riedy , S. K. Hong , J. D. Johnson , and N. Cohenca . 2011. “Quality of Life of Endodontically Treated Versus Implant Treated Patients: A University‐Based Qualitative Research Study.” Journal of Endodontics 37, no. 7: 903–909. 21689542 10.1016/j.joen.2011.03.026

[iej70018-bib-0013] Göransson, H. , T. Lougui , L. Castman , and L. Jansson . 2021. “Survival of Root Filled Teeth in General Dentistry in a Swedish County: A 6‐Year Follow‐Up Study.” Acta Odontologica Scandinavica 79, no. 5: 396–401. 33612053 10.1080/00016357.2021.1887513

[iej70018-bib-0014] Gõrduysus, M. O. , and M. G. Gõrduysus . 2000. “Endodontic Patient Profile of Hacettepe University, Faculty of Dentistry in Ankara, Turkey.” International Dental Journal 50, no. 5: 274–278. 15988886 10.1111/j.1875-595x.2000.tb00565.x

[iej70018-bib-0015] Hamasha, A. A. , and A. Hatiwsh . 2013. “Quality of Life and Satisfaction of Patients After Nonsurgical Primary Root Canal Treatment Provided by Undergraduate Students, Graduate Students and Endodontic Specialists.” International Endodontic Journal 46, no. 12: 1131–1139. 23560436 10.1111/iej.12106

[iej70018-bib-0016] Jonsson Sjögren, J. , T. Kvist , A. Eliasson , EndoReCo , and M. Pigg . 2019. “The Frequency and Characteristics of Pain and Discomfort Associated With Root Filled Teeth: A Practice‐Based Study.” International Endodontic Journal 52, no. 9: 1264–1273. 30980723 10.1111/iej.13124

[iej70018-bib-0017] Kebke, S. , H. Fransson , M. Brundin , and F. J. Mota de Almeida . 2021. “Tooth Survival Following Root Canal Treatment by General Dental Practitioners in a Swedish County—a 10‐Year Follow‐Up Study of a Historical Cohort.” International Endodontic Journal 54, no. 1: 5–14. 32871615 10.1111/iej.13392

[iej70018-bib-0018] Kirkevang, L. L. , D. Ørstavik , G. Bahrami , A. Wenzel , and M. Vaeth . 2017. “Prediction of Periapical Status and Tooth Extraction.” International Endodontic Journal 50, no. 1: 5–14. 26580306 10.1111/iej.12581

[iej70018-bib-0019] Laukkanen, E. , M. M. Vehkalahti , and A. K. Kotiranta . 2021. “Radiographic Outcome of Root Canal Treatment in General Dental Practice: Tooth Type and Quality of Root Filling as Prognostic Factors.” Acta Odontologica Scandinavica 79, no. 1: 37–42. 32529874 10.1080/00016357.2020.1773531

[iej70018-bib-0020] Leclaire, A. J. , A. E. Skidmore , J. A. Griffin Jr. , and F. S. Balaban . 1988. “Endodontic Fear Survey.” Journal of Endodontics 14, no. 11: 560–564. 3249195 10.1016/S0099-2399(88)80091-8

[iej70018-bib-0021] Leong, D. J. X. , and A. U. Yap . 2020. “Quality of Life of Patients With Endodontically Treated Teeth: A Systematic Review.” Australian Endodontic Journal 46, no. 1: 130–139. 31432613 10.1111/aej.12372

[iej70018-bib-0022] Lobb, W. K. , K. L. Zakariasen , and P. J. McGrath . 1996. “Endodontic Treatment Outcomes: Do Patients Perceive Problems?” Journal of the American Dental Association 127, no. 5: 597–600. 8642139 10.14219/jada.archive.1996.0271

[iej70018-bib-0023] Murillo‐Benítez, M. , J. Martin‐Gonzalez , M. C. Jimenez‐Sanchez , D. Cabanillas‐Balsera , E. Velasco‐Ortega , and J. J. Segura‐Egea . 2020. “Association Between Dental Anxiety and Intraoperative Pain During Root Canal Treatment: A Cross‐Sectional Study.” International Endodontic Journal 53, no. 4: 447–454. 31691312 10.1111/iej.13245

[iej70018-bib-0024] Newsome, P. R. , and G. H. Wright . 1999a. “A Review of Patient Satisfaction: 1. Concepts of Satisfaction.” British Dental Journal 186, no. 4: 161–165. 10205951 10.1038/sj.bdj.4800052

[iej70018-bib-0025] Newsome, P. R. , and G. H. Wright . 1999b. “A Review of Patient Satisfaction: 2. Dental Patient Satisfaction: An Appraisal of Recent Literature.” British Dental Journal 186, no. 4: 166–170. 10205952 10.1038/sj.bdj.4800053

[iej70018-bib-0026] Ng, Y. L. , V. Mann , and K. Gulabivala . 2011a. “A Prospective Study of the Factors Affecting Outcomes of Nonsurgical Root Canal Treatment: Part 1: Periapical Health.” International Endodontic Journal 44, no. 7: 583–609. 21366626 10.1111/j.1365-2591.2011.01872.x

[iej70018-bib-0027] Ng, Y. L. , V. Mann , and K. Gulabivala . 2011b. “A Prospective Study of the Factors Affecting Outcomes of Non‐Surgical Root Canal Treatment: Part 2: Tooth Survival.” International Endodontic Journal 44, no. 7: 610–625. 21366627 10.1111/j.1365-2591.2011.01873.x

[iej70018-bib-0028] Ng, Y. L. , V. Mann , S. Rahbaran , J. Lewsey , and K. Gulabivala . 2007. “Outcome of Primary Root Canal Treatment: Systematic Review of the Literature—Part 1. Effects of Study Characteristics on Probability of Success.” International Endodontic Journal 40, no. 12: 921–939. 17931389 10.1111/j.1365-2591.2007.01322.x

[iej70018-bib-0029] Nixdorf, D. R. , E. J. Moana‐Filho , A. S. Law , L. A. McGuire , J. S. Hodges , and M. T. John . 2010. “Frequency of Persistent Tooth Pain After Root Canal Therapy: A Systematic Review and Meta‐Analysis.” Journal of Endodontics 36, no. 2: 224–230. 20113779 10.1016/j.joen.2009.11.007 PMC2832800

[iej70018-bib-0030] Olsson, S. , J. Jonsson Sjögren , M. Pigg , et al. 2024. “Interventions in Root‐Filled Teeth Identified in General Dental Practice: A 6‐Year Longitudinal Observational Study.” International Endodontic Journal 57, no. 9: 1212–1227. 39302850 10.1111/iej.14079

[iej70018-bib-0031] Ørstavik, D. , K. Kerekes , and H. M. Eriksen . 1986. “The Periapical Index: A Scoring System for Radiographic Assessment of Apical Periodontitis.” Endodontics & Dental Traumatology 2, no. 1: 20–34. 3457698 10.1111/j.1600-9657.1986.tb00119.x

[iej70018-bib-0032] Peters, O. A. , ed. 2016. The Guidebook to Molar Endodontics. Springer.

[iej70018-bib-0033] Polycarpou, N. , Y. L. Ng , D. Canavan , D. R. Moles , and K. Gulabivala . 2005. “Prevalence of Persistent Pain After Endodontic Treatment and Factors Affecting Its Occurrence in Cases With Complete Radiographic Healing.” International Endodontic Journal 38, no. 3: 169–178. 15743420 10.1111/j.1365-2591.2004.00923.x

[iej70018-bib-0034] Reit, C. , G. Heden , and R. Milthon . 1993. “Endodontiskt Behandlingspanorama Inom Allmäntandvården.” Tandläkartidningen 85: 543–546.

[iej70018-bib-0035] Reit, C. , and T. Kvist . 1998. “Endodontic Retreatment Behaviour: The Influence of Disease Concepts and Personal Values.” International Endodontic Journal 31, no. 5: 358–363. 9823140 10.1046/j.1365-2591.1998.00170.x

[iej70018-bib-0036] Savolainen, N. , F. Frisk , and T. Kvist . 2025. “Is Root Canal Treatment and a Coronal Restoration of a Mandibular First Molar Cost‐Effective Compared to Extraction and an Implant‐Supported Crown? A Decision Analytic Approach.” Acta Odontologica Scandinavica 27, no. 84: 95–103. 10.2340/aos.v84.42894 PMC11926421 40014382

[iej70018-bib-0037] Segura‐Egea, J. J. , R. Cisneros‐Cabello , J. M. Llamas‐Carreras , and E. Velasco‐Ortega . 2009. “Pain Associated With Root Canal Treatment.” International Endodontic Journal 42, no. 7: 614–620. 19467050 10.1111/j.1365-2591.2009.01562.x

[iej70018-bib-0038] Silnovic, Z. , T. Kvist , and F. Frisk . 2023. “Periapical Status and Technical Quality in Root Canal Filled Teeth in a Cross Sectional Study in Jönköping, Sweden.” Acta Odontologica Scandinavica 81, no. 3: 249–254. 36098980 10.1080/00016357.2022.2121322

[iej70018-bib-0039] Sitzia, J. , and N. Wood . 1997. “Patient Satisfaction: A Review of Issues and Concepts.” Social Science & Medicine 45, no. 12: 1829–1843. 9447632 10.1016/s0277-9536(97)00128-7

[iej70018-bib-0040] Statens Beredning för medicinsk Utvärdering . 2010. “Rotfyllning. En Systematisk Litteraturöversikt.” Stockholm: Statens Beredning för medicinsk Utvärdering (SBU); 2010. SBU‐report; 203.

[iej70018-bib-0041] Strindberg, L. 1956. “The Dependence of the Results of Pulp Therapy on Certain Factors.” Acta Odontologica Scandinavica 14: 1–175.

[iej70018-bib-0042] Stull, D. E. , N. K. Leidy , B. Parasuraman , and O. Chassany . 2009. “Optimal Recall Periods for Patient‐Reported Outcomes: Challenges and Potential Solutions.” Current Medical Research and Opinion 25, no. 4: 929–942. 19257798 10.1185/03007990902774765

[iej70018-bib-0043] Torabinejad, M. , J. D. Kettering , J. C. McGraw , R. R. Cummings , T. G. Dwyer , and T. S. Tobias . 1988. “Factors Associated With Endodontic Interappointment Emergencies of Teeth With Necrotic Pulps.” Journal of Endodontics 14, no. 5: 261–266. 3251982 10.1016/S0099-2399(88)80181-X

[iej70018-bib-0044] Torabinejad, M. , W. Salha , J. L. Lozada , Y. L. Hung , and A. Garbacea . 2014. “Degree of Patient Pain, Complications, and Satisfaction After Root Canal Treatment or a Single Implant: A Preliminary Prospective Investigation.” Journal of Endodontics 40, no. 12: 1940–1945. 25305235 10.1016/j.joen.2014.08.022

[iej70018-bib-0045] Wigsten, E. , A. Al Hajj , P. Jonasson , EndoReCo , and T. Kvist . 2021. “Patient Satisfaction With Root Canal Treatment and Outcomes in the Swedish Public Dental Health Service. A Prospective Cohort Study.” International Endodontic Journal 54, no. 9: 1462–1472. 10.1111/iej.13548 33969501

[iej70018-bib-0046] Wigsten, E. , EndoReCo , and T. Kvist . 2022. “Patient Record Assessment of Results and Related Resources Spent During 1 Year After Initiation of Root Canal Treatment in a Swedish Public Dental Organization.” International Endodontic Journal 55, no. 5: 453–466. 35122276 10.1111/iej.13699 PMC9303384

[iej70018-bib-0047] Wigsten, E. , P. Jonasson , EndoReCo , and T. Kvist . 2019. “Indications for Root Canal Treatment in a Swedish County Dental Service: Patient‐ and Tooth‐Specific Characteristics.” International Endodontic Journal 52, no. 2: 158–168. 30107035 10.1111/iej.12998

[iej70018-bib-0048] Wigsten, E. , T. Kvist , P. Jonasson , EndoReCo , and T. Davidson . 2020. “Comparing Quality of Life of Patients Undergoing Root Canal Treatment or Tooth Extraction.” Journal of Endodontics 46, no. 1: 19–28.e1. 31843125 10.1016/j.joen.2019.10.012

